# Ionic Liquid Capsules as Flame-Retardant Additives for Emulsion Paint Systems

**DOI:** 10.3390/polym17050626

**Published:** 2025-02-26

**Authors:** Rong Ma, Bingqian Wu, Qingsheng Wang

**Affiliations:** 1 Artie McFerrin Department of Chemical Engineering, Texas A&M University, College Station, TX 77843, USA; marong1863@tamu.edu (R.M.); a1106844984@tamu.edu (B.W.); 2 Department of Chemical Engineering, University of Michigan, Ann Arbor, MI 48109, USA; 3 Department of Materials Science & Engineering, Texas A&M University, College Station, TX 77843, USA

**Keywords:** ionic liquids, graphene quantum dots, capsules, emulsion paint, flammability

## Abstract

To develop a highly efficient and environmentally friendly flame-retardant system, ionic liquids (ILs) have recently emerged as promising candidates. However, the addition of ILs into emulsion paint disrupts emulsion stability, leading to rapid demulsification due to electrostatic effects. Herein, IL–silica capsules were developed using a soft-template method. These capsules can be directly added to an acrylic emulsion paint system as flame-retardant additives. Incorporating 5 wt% IL–silica capsules into the system and coating it on fabric reduced flammability by 53% compared to untreated fabric. This work provides a novel and practical approach to enhance flame retardancy in emulsion paint systems without compromising their stability.

## 1. Introduction

The enormous amount of damage inflicted by fire each year across the world, including major property loss, human deaths, and injuries, has raised awareness and highlighted the critical need for efficient fire-resistance technology and materials [1,2]. As a result, flame-retardant chemicals have been added to a wide range of consumer products and used as treatments on materials to prevent fires, limit fire spread, and reduce fire damage [3,4,5]. For example, flame retardants can be added to paint and coatings, which can then be applied to a variety of combustible or non-combustible surfaces to dramatically reduce the risk and damage from fire in a range of settings from buildings to vehicle interiors. However, conventionally effective halogen-based flame retardants such as polybrominated diphenyl ethers (PBDEs) pose health and environmental hazards due to their tendency to migrate into surroundings and living tissues; thus, there has been an ongoing effort to ban and replace toxic flame retardants with greener alternatives [6,7,8].

To replace halogen-based flame retardants and avoid toxic volatile substances during the combustion process, flame retardants with silicon, phosphorus, or nitrogen element have been applied [9,10,11]. Phosphorous flame retardants generate free phosphorus radicals during the combustion process, which leads to the quenching of free radicals of volatile products and the cessation of the combustion process [12,13].

Ionic liquids (ILs) are known as organic salts and have been used as the most promising solvents and catalysts due to their wide liquid range [14,15,16]. They have also been receiving increasing attention recently as a type of green flame-retardant agent due to their negligible vapor pressure, unique designability, low flammability, high thermal stability, and low toxicity. There are some research works reporting that ILs have a special structure and can be used as a synergist to impart flame retardancy to different types of polymer materials. For example, a phosphorus-containing ionic liquid ([PCMIM]Cl) was used with ammonium polyphosphate (APP) together as a flame retardant for polypropylene [14]. This shows that the addition of [PCMIM]Cl to this intumescent system is beneficial for the formation of an excellent char layer and the inhibition of the combustion process. Similarly, a type of N-methylimidazole sulfonate ionic liquid ([Dmim]Tos) with a DOPO structure, containing phosphorous and imidazole, was designed and has been used as a flame retardant for epoxy (EP) [17]. Due to the excellent flame-retardant efficiency and good affinity with EP, the prepared EP system exhibits high fire safety and good mechanical properties. Another study successfully mixed waterborne polyurethane dispersion with aqueous [Dmim]Tos solution with 6 wt% loading and achieved a decrease of 46% and 41.5% in the peak heat release rate (pHRR) and total heat release (THR) when compared to control samples with 0 wt% IL loading [18].

Emulsion paint is a type of water-based acrylic paint invented during the period of World War II because of linseed oil shortages. Due to it being odorless, non-toxic emulsion paint quickly overshadowed solvent-based paint following its invention and gradually became the standard paint type for houses today [19]. In emulsion paint, water is used as a solvent in which the binder, pigment, and additives are dispersed. The binder is a substance that forms a dry coating or film, and is typically a polymer material, such as acrylic resins, epoxies, polyurethane, etc. [19]. However, these organic components in emulsion paint are prone to combustion when exposed to high temperatures or open flames, posing potential fire hazards. With increasing fire protection requirements, introducing flame-retardant properties into emulsion paint has become a critical research focus. In our preliminary test, when ILs were directly dispersed in emulsion paint, it showed signs of instability from creaming and coalescence immediately after mixing and quickly separated into two distinctive phases. Also, ILs have the disadvantage of poor water resistance if put into the oil-in-water emulsion system directly [20]. In order to test the potential application of ILs as a flame retardant in emulsion paint, a method must be developed to overcome the challenge of emulsion stability.

Here, IL capsules used as flame-retardant agents were developed by using the soft-template method. The amphiphilic graphene quantum dots synthesized based on our previous work were used to stabilize IL-in-water Pickering emulsions [21,22]. Then, ILs were encapsulated by forming a silica shell. These prepared ILs capsules were mixed with emulsion paint directly as flame-retardant additives. The flame retardancy of the emulsion paint system with ILs capsules was evaluated by using a microscale combustion calorimeter.

## 2. Materials and Methods

### 2.1. Materials

Commercial white acrylic emulsion paint with a density of 1.3 g/mL was obtained from Home Depot (Bryan, TX, USA). BR-S13 phosphorous flame-retardant ionic liquid is a type of commercial ionic liquid that was obtained from Inovia Materials LLC (Boulder, CO, USA). Citric Acid (CA, 98%) from Sigma-Aldrich (St. Louis, MO, USA), octadecylamine (ODA) from Acros Organics (Waltham, MA, USA), and dodecylamine (DA, 98%) from TCI Development Co., Ltd. (Philadelphia, PA, USA). were used to prepare amphiphilic GQDs. Tetraethyl Orthosilicate (TEOS) was obtained from Sigma-Aldrich (St. Louis, MO, USA), and 3-aminopropyltriethoxysilane (APTES, 98%) was purchased from TCI Development Co., Ltd. (Philadelphia, PA, USA). Tween 80 surfactant was obtained from Sigma-Aldrich (St. Louis, MO, USA). All purchased chemicals were used directly after receiving them.

### 2.2. Fabrication of IL–Silica Microcapsules

Two types of amphiphilic graphene quantum dots (C12-GQDs and C18-GQDs) were synthesized based on our previous work [21,22]. In brief, CA and DA or ODA used as reactants were mixed in a vial by using a vortex mixture. The mass ratio of CA to DA or ODA is 2:1. After mixing, the vial was placed in an oven to react at 200 °C in air for 2.5 h, where brown crude products were obtained. After cooling down, the crude products were purified to remove any unreacted reagents. Finally, amphiphilic nanoplatelets of GQD functionalized with octadecyl (C18-GQDs) or dodecyl (C12-GQDs) were obtained.

**Figure 1:**
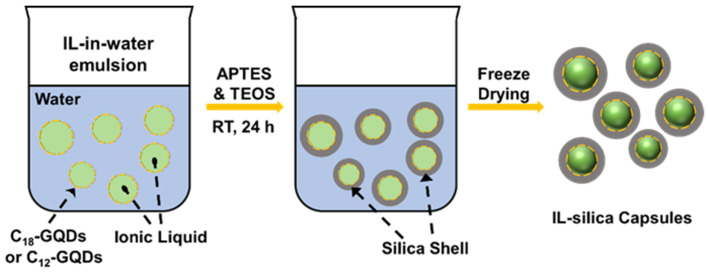
The preparation of C12-IL–silica capsules or C18-IL–silica capsules.

The IL–silica capsule preparation process is shown in Figure 1. To encapsulate IL within silica shells, a stable IL/W Pickering emulsion was obtained first. The commercial IL BR-S13, a type of phosphorous IL, was chosen as the oil phase, and DI water was chosen as the aqueous phase. First, C12-GQD or C18-GQDs were dispersed in DI water to obtain the 10 mL of 0.05 wt% nanoplatelet dispersion. Then, 1 mL of IL was added to the nanoplatelet dispersion and vortexed for a short period. Then, the mixture system was homogenized using a sonication tip for 15 min, and stable IL/W Pickering emulsions were obtained. After the samples cooled down to room temperature, 0.8 mL of TEOS was added to the emulsion drop by drop, followed by 0.2 mL of APTES, while the emulsion was stirred. Samples were stirred for 24 h at room temperature, and white solid particles were observed in each sample at the end of the reaction period. The samples were washed with DI water, centrifuged 3 times washing, and then dried by freeze-drying for 12 h. Additionally, the TEOS and APTES mixture was added into another vial of 10 mL DI water, which served as a control sample and was not loaded with IL, named as neat silica. Three samples prepared with different amphiphilic GQDs were labeled C12-IL–silica capsules and C18-IL–silica capsules.

### 2.3. Characterizations

The chemical structure was characterized by Fourier transform infrared spectroscopy (FTIR, Thermo Nicolet 380) using the ATR mode. The thermal stability of each sample was investigated using a thermo-gravimetric analyzer (TGA Q500) at 20.00 °C/min ramp rate from 30 °C to 700 °C in a pure nitrogen atmosphere. The morphology of IL-in-silica capsules was observed using field emission scanning electron microscopy (FE-SEM, JSM-7500F, JEOL, Akishima City, Japan). The mapping of periodic elements in capsules was achieved using energy dispersive X-ray spectroscopy (EDS, Oxford EDS system, Oxford Instruments, Carteret, NJ, USA). For SEM and EDS testing, all samples were dried in an oven at 40 °C after washing with DI water.

The flammability parameters of IL-in-silica capsules in emulsion paint were determined using a micro combustion calorimeter (MCC, ASTM D7309). MCC tests were conducted on samples of cotton fabric treated with diluted paint mixed with different concentrations of IL capsules. All treatment solutions were made from 20 *v*/*v* % paint and 80 *v*/*v* % water solution, and then neat silica (control), C12-IL–silica capsules, or C18-IL–silica capsules were added to the diluted paint at different wt% with reference to the weight of paint. Then, all solution samples were vortexed for 20 s to make a homogenous solution. Cotton fabrics were prepared by cutting them into uniform dimensions of 10 × 20 mm, and then all fabric samples were submerged into different treatment solutions for 3 min. Afterward, each fabric sample was removed from its solution and dried at room temperature for 48 h.

## 3. Results and Discussion

### 3.1. Properties of IL–Silica Capsules

**Figure 2:**
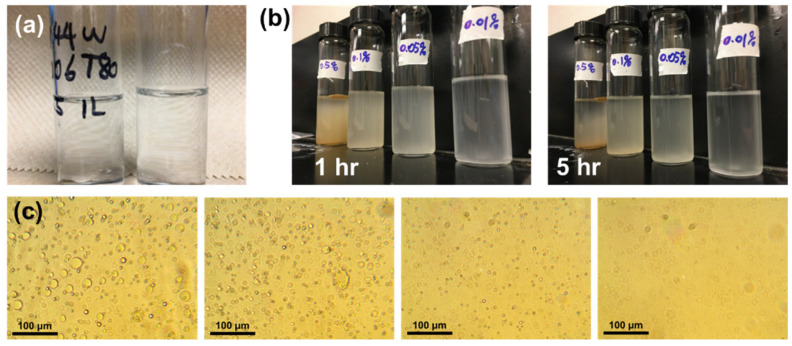
Emulsification behavior of IL/W using (**a**) Tween 80 (left) and Tween 20 (right) surfactants and (**b**) amphiphilic GQDs with concentration 0.5, 0.1, 0.05, 0.01 *wt*% (from left to right) to stabilize for 1 h and 5 h. (**c**) Microscope images of IL/W emulsions with different concentrations of C12-GQDs: 0.5, 0.1, 0.05, and 0.01 *wt*% (from left to right).

To prepare IL microcapsules, the IL/W emulsion was created first. The different emulsion behaviors of the IL/W emulsion with different types of surfactants are shown in Figure 1. Commercial non-ionic surfactants Tween 20 and Tween 80 were used as the emulsifier, but none of them resulted in successful emulsification (Figure 2a). However, from Figure 2b, it can be observed that, when using amphiphilic GQDs as emulsifiers, they can form a stable IL/W Pickering emulsion and are stable for 5 h. From the optical microscope images of the prepared Pickering emulsions (Figure 2c), they show the presence of micro-sized IL droplets in water. They also show that the emulsions become less stable over time (Figure 2c), as more IL droplets appear to aggregate at the bottom of the samples with decreasing C12-GQD concentration. Considering suitable emulsion stability and droplet sizes for the capsule preparation process, the 0.05 wt% amphiphilic GQDs were chosen to prepare IL capsules for further studies.

**Figure 3:**
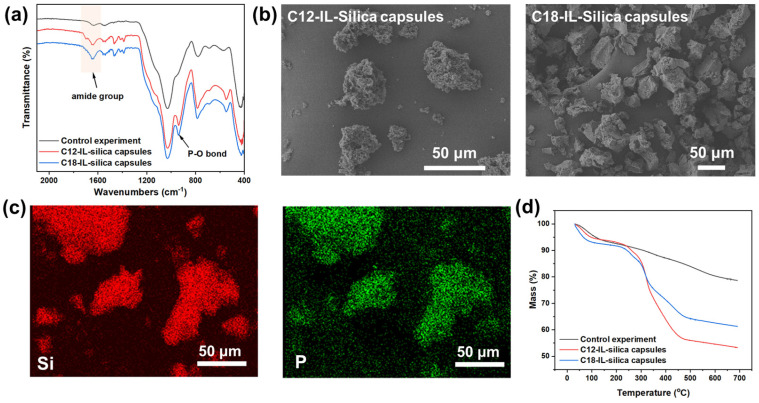
Characterization of prepared IL–silica capsules. (**a**) ATR−FTIR, (**b**) SEM image, (**c**) EDS mapping of Si and P, and (**d**) TGA curves of IL−silica capsules.

From the ATR-FTIR spectra (Figure 3a), the amide groups ranging from 1600 to 1800 cm^−1^ demonstrate the incorporation of amphiphilic GQDs [21]. At close to 940 cm^−1^, the peak indicates a P-O bond [23], which suggests the incorporation of phosphorous ILsBR-S13 incorporation in the capsules. The morphologies of the prepared microcapsules (C12-IL–silica capsules and C18-IL–silica capsules) were investigated by using SEM, as shown in Figure 3b. C12-IL–silica capsule images show non-spherical silica-shelled capsules loaded with ILs. While C18-IL–silica capsule samples show capsules that are similar in shape and size, the average size of the prepared capsules is 43 ± 7 μm. This shape of the prepared capsules might be further controlled by altering the TEOS/APTES ratios and reaction time [24]. To further confirm the encapsulation of phosphorous ILs, EDS mapping was performed to determine the element composition and element distribution of C12-IL–silica capsules. In Figure 3c, the locations of silica (red) and phosphorous (green) are overlapped, which can demonstrate that the phosphorus IL was encapsulated in the prepared IL–silica capsules.

The thermal stability of microcapsules was determined by using a TGA method, shown in Figure 3d. Based on the TGA curve, the neat silica samples show a two-step mass loss profile, while the IL-loaded capsules show a three-step profile. The first step in the range of 50–100 °C corresponds to the evaporation of physically absorbed water in the samples, and the second step starting from 150 °C can be attributed to the hydrolysis of Si-OH and Si-OC2H5 [25,26]. For TGA curves of IL–silica capsules, the third step represents the weight loss from the decomposition of the emulsifiers GQDs and ILs. This step of weight loss ranges from 250 °C to 450 °C, and within this range, C12-IL–silica capsules experience a weight loss of 37%, and C18-IL–silica capsules experience a weight loss of 27.6%.

### 3.2. Stability of Flame-Retardant Paint Systems

**Figure 4:**
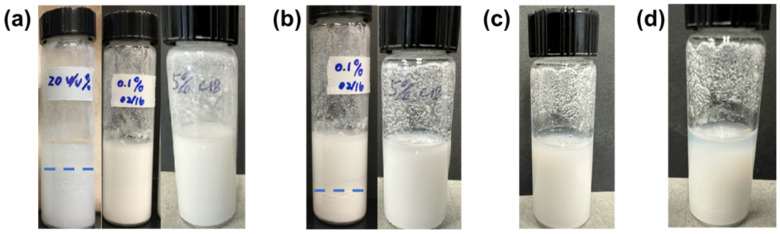
The stability of emulsion paint systems. (**a**) IL, IL/W Pickering emulsion, and C12-IL–silica capsules in paint (from left to right) 5 min after mixing; (**b**) IL/W Pickering emulsion (left) and C12-IL–silica capsules in paint (right) 3 h after mixing; C12-IL–silica capsules in paint (**c**) 24 h and (**d**) 48 h after mixing.

The stability of the flame-retardant paint system affects their applications. Figure 4 shows a comparison of the state of the emulsion paint after the addition of the respective components. The mixture of ILs and paint resulted in almost immediate demulsification upon the addition of the IL within 5 min (vial on left in Figure 4a), as the sample separated itself into two distinctive phases of different opacity. Shadows of small dents near the surface of the glass vial indicate that the bottom phase contains mostly small grains of solid acrylic resin. The mixture of IL/W Pickering emulsion and acrylic paint shows a mostly uniform color and texture (the second vial in Figure 4a), unlike in the first vial. However, after 3 h, the samples show a similar demulsification process, as shown in the vial on the left in Figure 4b. This could be explained by the fact that the amphiphilic GQDs cannot tightly cover the entire surface of IL droplets to avoid release, so the impact of ILs on emulsion paint remains the same but at a reduced rate. The mixture of IL–silica capsules in the emulsion paint system remains mostly uniform within 24 h (right vials in Figure 4a–c), and phase separation becomes distinctive after 48 h (Figure 4d), which shows that the encapsulation method does have a positive effect on improving the stability of the emulsion paint system.

### 3.3. Flame-Retardant Properties of Emulsion Paint Systems

**Figure 5:**
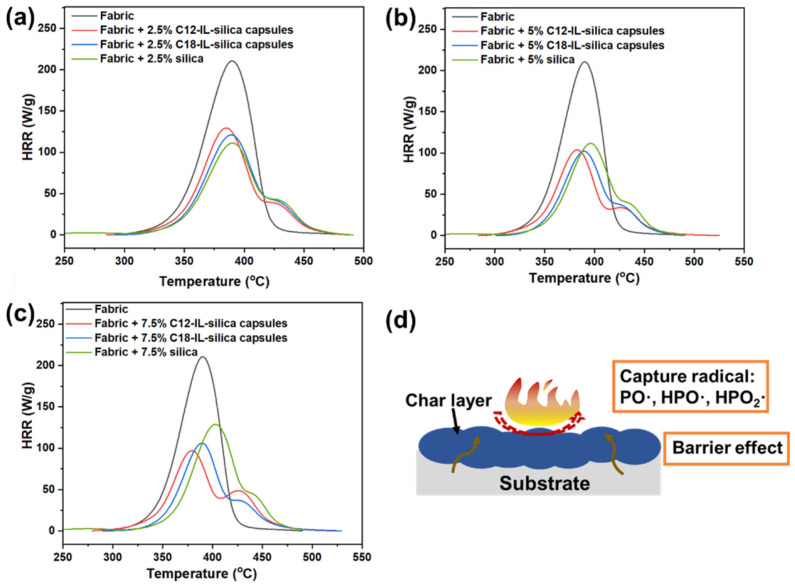
MCC result of emulsion paint with loadings of (**a**) 2.5 wt%, (**b**) 5 wt%, and (**c**) 7.5 wt% of IL–silica capsules. (**d**) Mechanism of IL–silica capsules as flame-retardant additives in emulsion coating system.

The microscale combustion calorimeter (MCC) analysis shown in Figure 5 and Table 1 presents a comparison of flammability parameters between blank cotton fabric and fabrics coated in emulsion paint with different loadings of silica (C12-IL–silica capsules and C18-IL–silica capsules). In these experiments, the emulsion paints with a loading of 2.5 wt%, 5 wt%, and 7.5 wt% of IL–silica capsules were used to investigate the flammability. With more IL–silica capsules, the peak heat release rate (pHRR) decreases, but the peak temperatures are almost the same. As shown in Figure 5a, for the 2.5 wt% IL–silica loading in the emulsion paint system, there is a 47% decrease in the pHRR, which is due to silica’s effective flame-retardant effects. However, there is no further improvement in flame retardancy after the incorporation of limited ILs. When adding 5 wt% additives into the emulsion paint (Figure 5b), the pHRR values are further decreased by around 9% with the incorporation of ILs for C12-IL–silica capsules and C18-IL–silica capsules. When adding 7.5 wt% C12-IL–silica capsules (Figure 5c), these samples show a 53% reduction in the pHRR and a 38% decrease in total heat release (THR) compared with blank fabric.

The proposed mechanism is that silica capsules decompose and release IL under high temperatures [27]. After the degradation of phosphorus ILs, this results in the formation of a phosphorus-rich residue layer that can inhibit mass and heat transfer [17,28]. Furthermore, phosphorus-containing free radicals are released into the gaseous phase to quench free radicals generated in the combustion process and terminate the chain reaction [17,28]. These results demonstrate that the flame-retardant performance was improved after the incorporation of ILs into capsules.

## 4. Conclusions

In this work, we developed IL–silica capsules as flame-retardant agents for emulsion paint systems by using the Pickering emulsion template method. These IL–silica capsules maintain the stability of the emulsion paint system when mixed, while the system separates into two phases when adding ILs directly. The prepared capsules incorporate phosphorus ILs and silicon shells, which have been approved by an element analysis test. The flame-retardant properties of the emulsion pain system with IL–silica capsules are significantly improved and can be controlled by adjusting the types of IL–silica capsules and their loading amount. Notably, the emulsion paint with 5 wt% C12-IL–silica exhibits the lowest pHRR, achieving the best flame retardancy. This work demonstrates a feasible concept to create non-toxic and environmentally friendly flame-retardant additives and enhance the fire-resistant properties of emulsion paints.

## Figures and Tables

**Table 1:** Flammability parameters of samples for MCC test.

Sample	Peak HRR (W/g)	Total HR (kJ/g)	Temperature (C)
Fabric	210.6	10.8	390.2
Fabric + 2.5% silica	111.2	7	390
Fabric + 5% silica	111.6	6.9	391.9
Fabric + 7.5% silica	128.9	8	391.2
Fabric + 2.5% C12-IL–silica capsules	129.3	7.5	385
Fabric + 5% C12-IL–silica capsules	103.7	6.3	382.3
Fabric + 7.5% C12-IL–silica capsules	97.13	6.7	379.1
Fabric + 2.5% C18-IL–silica capsules	121	7.2	389
Fabric + 5% C18-IL–silica capsules	102	6.1	389.7
Fabric + 7.5% C18-IL–silica capsules	106	6.3	389.1

## Data Availability

The original contributions presented in this study are included in the article. Further inquiries can be directed to the corresponding author.
